# An antibacterial coated polymer prevents biofilm formation and implant-associated infection

**DOI:** 10.1038/s41598-021-82992-w

**Published:** 2021-02-11

**Authors:** Hiroko Ishihama, Ken Ishii, Shigenori Nagai, Hiroaki Kakinuma, Aya Sasaki, Kenji Yoshioka, Tetsuya Kuramoto, Yuta Shiono, Haruki Funao, Norihiro Isogai, Takashi Tsuji, Yasunori Okada, Shigeo Koyasu, Yoshiaki Toyama, Masaya Nakamura, Mamoru Aizawa, Morio Matsumoto

**Affiliations:** 1 Department of Orthopaedic Surgery, Keio University School of Medicine, 35 Shinanomachi Shinjuku, Tokyo, 160-8582 Japan; 2 Department of Microbiology and Immunology, Keio University School of Medicine, Tokyo, Japan; 3 Department of Pathology, Keio University School of Medicine, Tokyo, Japan; 4 Department of Orthopaedic Surgery, School of Medicine, International University of Health and Welfare (IUHW), 852 Hatakeda, Narita City, Chiba 286-8520 Japan; 5 Department of Applied Chemistry, School of Science and Technology, Meiji University, Ikuta, Kanagawa Japan; 6 Kanagawa Academy of Science and Technology (KAST), Kawasaki, Kanagawa Japan; 7 Core Research for Evolutional Science and Technology (CREST), Japan Science and Technology Agency (JST), Tokyo, Japan; 8 Department of Molecular Immunology, Graduate School of Medical and Dental Sciences, Tokyo Medical and Dental University, Tokyo, Japan; 9 Department of Pathology and Oncology, School of Medicine, Juntendo University, Bunkyo, Tokyo Japan; 10 Laboratory for Immune Cell Systems, RIKEN Center for Integrative Medical Sciences (IMS), Yokohama, Kanagawa Japan

**Keywords:** Skeleton, Diseases

## Abstract

To prevent infections associated with medical implants, various antimicrobial silver-coated implant materials have been developed. However, these materials do not always provide consistent antibacterial effects in vivo despite having dramatic antibacterial effects in vitro, probably because the antibacterial effects involve silver-ion-mediated reactive oxygen species generation. Additionally, the silver application process often requires extremely high temperatures, which damage non-metal implant materials. We recently developed a bacteria-resistant coating consisting of hydroxyapatite film on which ionic silver is immobilized via inositol hexaphosphate chelation, using a series of immersion and drying steps performed at low heat. Here we applied this coating to a polymer, polyetheretherketone (PEEK), and analyzed the properties and antibacterial activity of the coated polymer in vitro and in vivo. The ionic silver coating demonstrated significant bactericidal activity and prevented bacterial biofilm formation in vitro. Bio-imaging of a soft tissue infection mouse model in which a silver-coated PEEK plate was implanted revealed a dramatic absence of bacterial signals 10 days after inoculation. These animals also showed a strong reduction in histological features of infection, compared to the control animals. This innovative coating can be applied to complex structures for clinical use, and could prevent infections associated with a variety of plastic implants.

## Introduction

Various biomaterials with a variety of shapes and properties are used in the manufacture of implantable medical devices. Such devices generally consist of steel, titanium alloy, cobalt chrome, and non-metal materials including ceramics, polyethylene, polyetheretherketone (PEEK), polymethylmethacrylate (PMMA) cements, and others. As implant use is increasing, the incidence of implant-associated infection (IAI), a serious complication, is also on the rise^1–3^. The treatment of IAI often requires long-term antibiotic administration, but if biofilm is present, the drugs may fail to penetrate the biofilm and kill the component organisms; even activated phagocytes cannot kill the bacteria in biofilms^4^. Thus, attempts to treat patients who are adversely affected by biofilm formation on or near implants often fail^5–8^. Because chemotherapy cannot completely resolve biofilm-associated infections, additional surgery is often necessary to wash out, drain, or remove the implant. Although these infections are usually caused by relatively common indigenous or attenuated skin bacterial pathogens^6^, some involve multidrug-resistant bacteria that can be quite intractable.

To prevent IAI, various metal implants with antimicrobial effects have been developed in recent years^9^. Silver coating on non-metallic implants has also been reported, with some clinical use^10–13^. Silver is thought to exert antibacterial effects by mediating the generation of reactive oxygen species in the presence of oxygen. To maintain its antibacterial activity in vivo, the silver needs to remain in an ionic state and to be stably bound to the implant surface. Plasma-spraying methods involve extremely high temperatures that make them inappropriate for various non-metal materials. We recently developed a bacteria-resistant coating consisting of hydroxyapatite film, on which ionic silver is immobilized via inositol hexaphosphate chelation using a low-heat immersion process^14^. This coating onto the metal demonstrates remarkable antibacterial activity both in vitro and in vivo^14^. On the other hand, the synthetic resin PEEK is widely used for orthopedic devices such as spinal interbody cages and components for joint arthroplasty. We have also developed a preliminary bacteria-resistant coating on PEEK^15^. However, it is generally difficult to process any coatings on PEEK because of its chemical stability, and there had been no previous report on its antibacterial processing.

Bioluminescence imaging (BLI) enables the real-time and non-invasive sequential monitoring of gene expression and cell growth in vivo^16–19^. In this method, cells that emit a constant bioluminescent signal can be detected through the tissues of a live animal using an ultrasensitive, cooled, charge-coupled device (CCD) camera. The family of luciferase enzymes consists of proteins that can generate visible light in the presence of oxygen and, generally, ATP as a source of energy. This approach has been proven useful in various studies such as oncology^20^, endocrine disruptors^21^, metabolism^22^, regenerative medicine^16,17^, immunology^23^, and infections^19,24–27^. We recently established a quantitative musculoskeletal infection model using BLI, in which we visualized and quantified bacterial growth in the mouse gluteus muscle in vivo^19^. We were able to monitor the infectious process throughout the course of the disease, in both the acute and chronic phases, without sacrificing the animals. This animal model may be a powerful tool for evaluating the pathophysiology of infection and the efficacy of new antibacterial drugs and implants. The purposes of the present study were to evaluate an optimal processing of our bacteria-resistant coating for implanted biomedical polymers and to analyze its antibacterial effect both in vitro and in vivo.

## Results

### Optimization of the concentrated sulfuric acid immersion time

**Figure 1:**
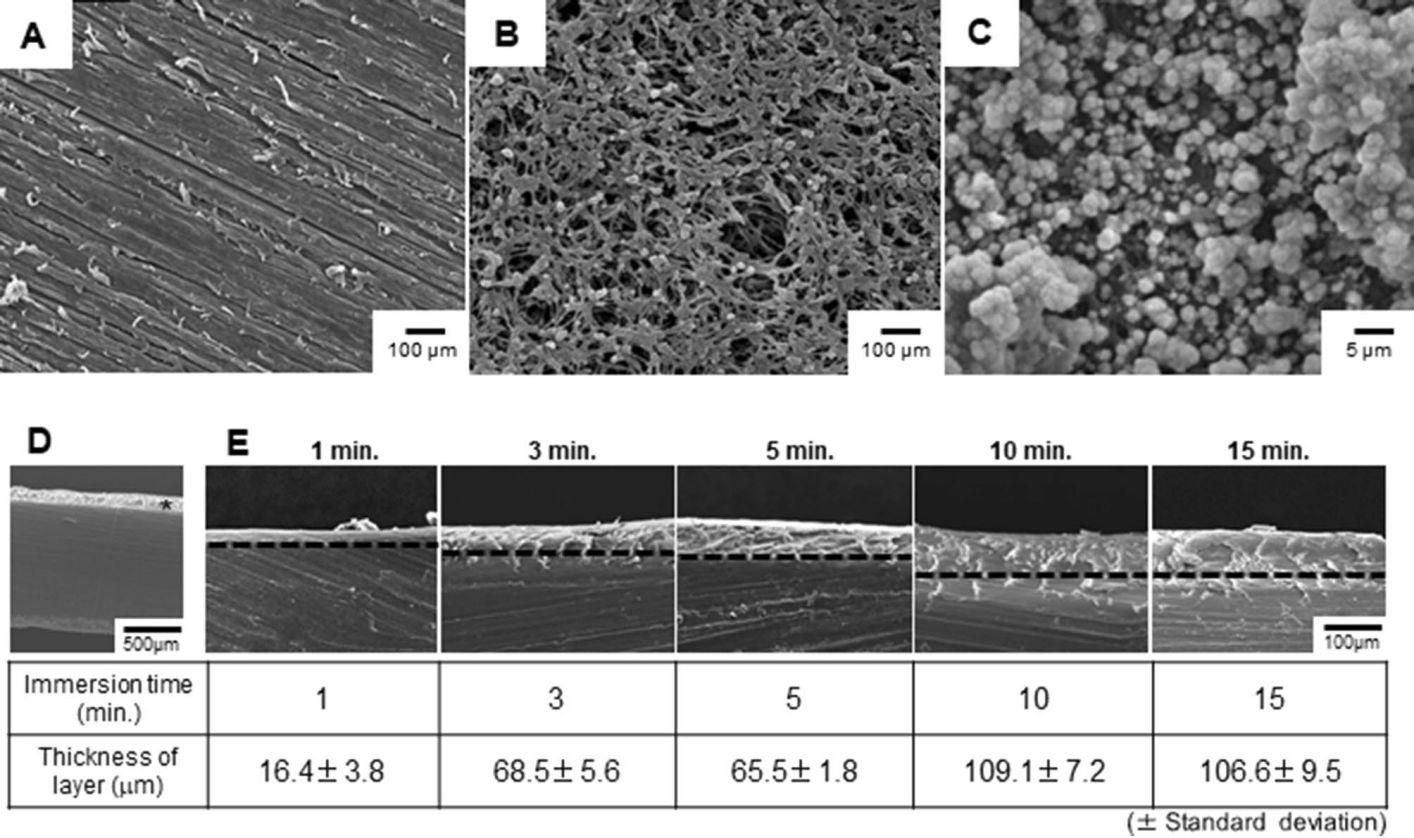
SEM images of untreated and H_2_SO_4_-treated PEEK and of PEEK-Ag^+^. The surface of pure polyetheretherketone (PEEK) (**A**) was immersed in 98% concentrated sulfuric acid (H_2_SO_4_) for 10 min (**B**), after which a porous configuration was observed on the surface by SEM. Hydroxyapatite (HAp) aggregates were homogeneously coated on the PEEK coated with immobilized Ag^+^ ions (PEEK-Ag^+^) (**C**). The H_2_SO_4_ modification of the PEEK surface was performed for different periods of time. Vertical sections of the PEEK plates are shown in (**D**) and (**E**). Asterisk reveals the reaction layer caused by H_2_SO_4_ treatment. After 10 min of treatment in a 100-µm depth, the thickness of the modification stayed about the same.

Plates of pure PEEK (Fig. 1A) were immersed in 98% concentrated sulfuric acid (H_2_SO_4_) for various durations, and the modification of the plates’ surface was observed by scanning electron microscopy (SEM) (Fig. 1B,D,E). With an immersion time of 1, 3, and 5 min, the average thickness of the modification layer was 16.4 ± 3.8, 68.5 ± 5.6, and 65.5 ± 1.8 µm, respectively. With 10- and 15-min-immersion times, the thickness of the reaction layer was 109 ± 7.2 and 106.6 ± 9.5 µm, respectively. With treatments over 10 min, the thickness of the modification did not change very much (approximately 100 µm); therefore, a 10-min-immersion time appeared to be sufficient for the surface modification. The biomechanical analysis of these treated PEEK cages is described below (Results Section: Validation of biomechanical stability).

### Material properties of the coating formed on the PEEK

**Figure 2:**
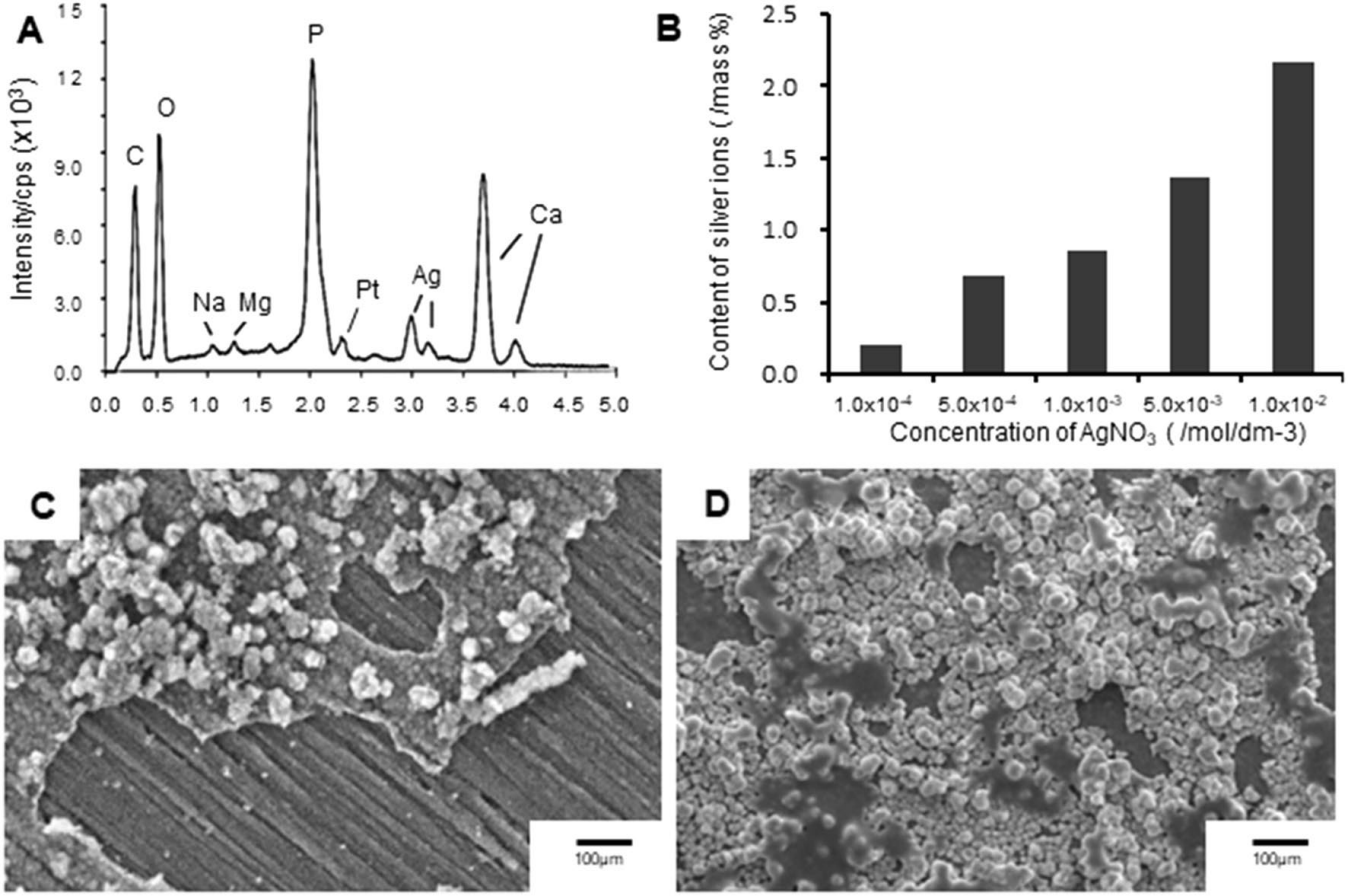
Microstructure and component analyses of the PEEK-Ag^+^. The surface of the PEEK-Ag^+^ was qualitatively assessed using energy dispersive X-ray analysis (EDX) (**A**). The film consisted of Ca, P, O, C, and Ag. Inductively coupled plasma atomic emission spectrometry (ICP-AES) indicated that the Ag^+^ ion content in the HAp film was dependent on the AgNO_3_ concentration used (**B**). A peeling test showed that the HAp adhered poorly to the Ag^+^-coated PEEK prepared without H_2_SO_4_ (**C**), while the H_2_SO_4_-immersed PEEK-Ag^+^ adhered strongly enough to withstand being peeled off with tape (**D**).

A SEM image of the microstructure of the PEEK surface without H_2_SO_4_ treatment is shown in Fig. 1A. After being immersed in H_2_SO_4_ solution for 10 min, the PEEK surface had a porous configuration (Fig. 1B); the pores had a diameter of 1–5 µm. A typical SEM image of the PEEK coated with immobilized Ag^+^ ions (PEEK-Ag^+^) surface is shown in Fig. 1C. A hydroxyapatite (HAp) aggregates were homogeneously coated onto the acid-treated PEEK substrate (Fig. 1C). Qualitative analysis of the PEEK-Ag^+^ surface using energy dispersive X-ray analysis (EDX) showed that the film consisted of Ca, P, O, C, and Ag (Fig. 2A). The presence of Pt was due to sputtering prior to the SEM observation. These results indicated that HAp aggregates were precipitated onto the PEEK substrate, and that Ag^+^ ions were immobilized on the HAp film by the chelate-bonding ability of inositol phosphate 6 (IP6). The composition of the Ag^+^ immobilized on the HAp film formed on the PEEK substrate was analyzed by inductively coupled plasma atomic emission spectrometry (ICP-AES). The quantitative analysis indicated that the Ag^+^ ion content in the HAp film increased with the concentration of silver nitrate solution used (Fig. 2B).

We previously assessed the cytotoxicity of this coating prepared with different amounts of silver nitrate (AgNO_3_) (1, 5, 10 mmol·dm^−3^) based on the ISO standard 10993-5 in mouse fibroblast L-929 cells [American Type Culture Collection (ATCC) CCl-1]^14^. Briefly, cells were seeded in the wells of cell-culture plates and incubated until approximately 80% confluent. The cell-culture medium was replaced by unfiltered medium into which a substrate treated with the coating had been extracted. The cells were then cultured at 37 °C in 5% CO_2_ for 3 days. The toxicity of the coating treated with 5 mmol·dm^−3^ AgNO_3_ was less than 25%, which is acceptable according to Food and Drug Administration (FDA) standards. Therefore, the coating prepared with 5 mmol·dm^−3^ AgNO_3_ was used for our in vivo experiments^14^. The resulting Ag spectra contained various Ag chemical states, such as Ag (0), Ag_2_O (+1), Ag_3_PO_4_ (+1), and Ag-unknown^14^.

Peeling tests showed extensive film defects of the HAp in Ag^+^-coated PEEK prepared without H_2_SO_4_ treatment (Fig. 2C), while the H_2_SO_4_-immersed PEEK-Ag^+^ had good adhesion that could withstand the peeling tape (Fig. 2D). These results suggested that the porous surface of the H_2_SO_4_-treated PEEK substrate was an effective scaffold for holding the HAp.

### Antibacterial effect in vitro

**Figure 3:**
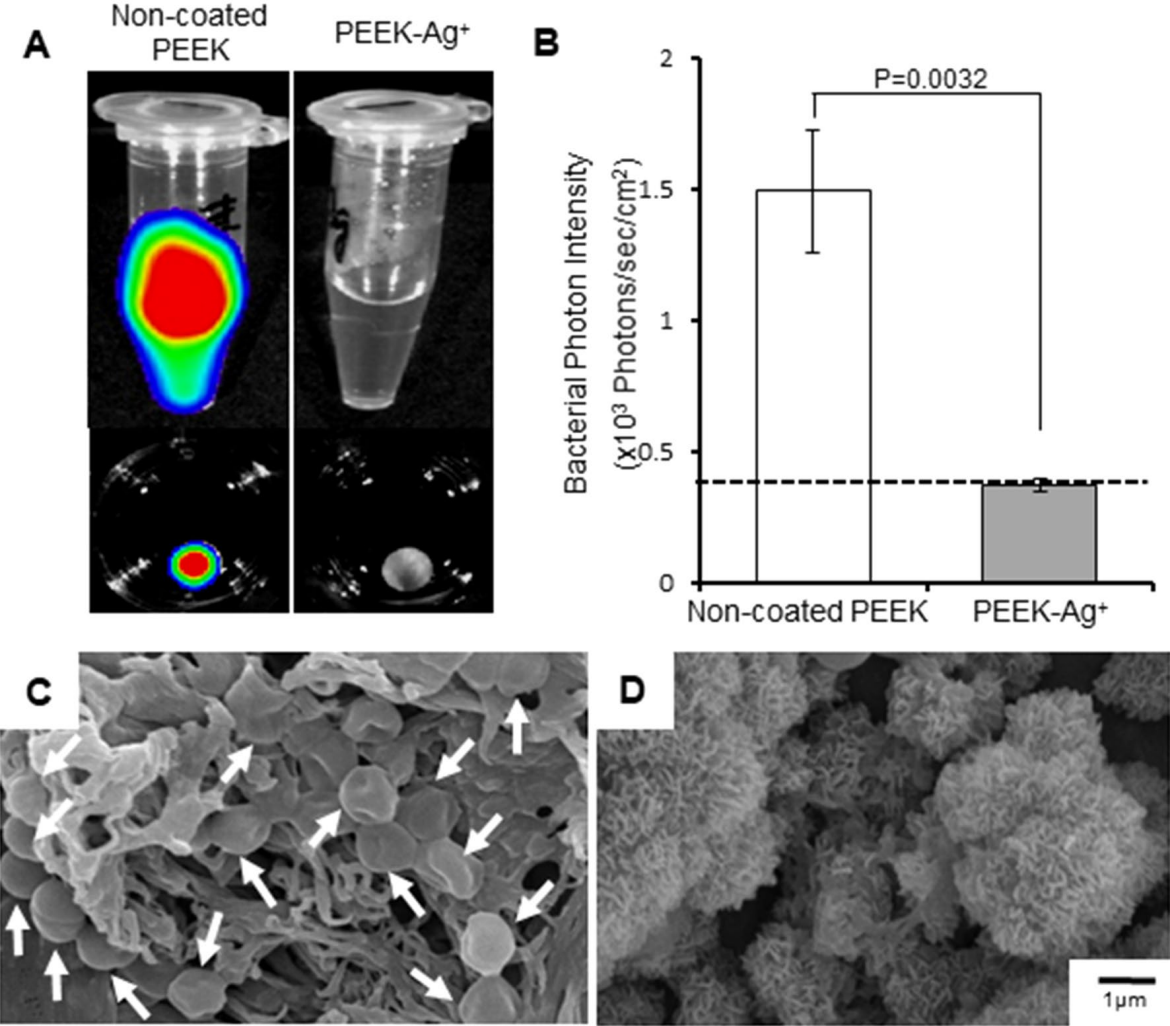
Antibacterial effect in vitro. Strong bacterial bioluminescent signals were detected in extracted medium from the non-coated PEEK plate and its surface (**A**, left). In contrast, no bacterial signals in the extracted medium or the surface of the removed PEEK-Ag^+^ plate were detected (**A**, right). The signals in the non-coated PEEK samples were significantly greater than in the PEEK-Ag^+^ ones (*p* = 0.0032) (**B**). These observations suggested that the bacteria were not alive in the PEEK-Ag^+^ group. SEM analysis revealed *S. aureus* spherical bodies (arrows) and bacterial biofilm on the surface of the non-coated PEEK plate (**C**), but not on the PEEK-Ag^+^ plate (**D**). Results shown are means ± standard deviations (SD).

Non-coated PEEK (control) and PEEK-Ag^+^ plates were immersed into 500 µl of medium containing 5 × 10^7^ colony-forming units (CFU) of bioluminescent Staphylococcus aureus (*S. aureus*) at 37 °C for 24 h. The PEEK plates were then removed from the medium and washed with phosphate buffered saline (PBS). Strong bacterial signals were detected in the extract medium from the non-coated PEEK plate and its surface. In contrast, no bacterial bioluminescent signals were detected in the extract medium or the surface of the removed PEEK-Ag^+^ plate, suggesting that the bacteria were not alive. The mean photon intensity (PI) of the surface of the PEEK-Ag^+^ (0.376 ± 0.023 × 10^3^ PI) was significantly lower than that of the non-coated PEEK (1.500 ± 0.235 × 10^3^ PI, *p* = 0.0032, Fig. 3A,B). The average PI of 0.376 on the surface of the PEEK-Ag^+^ plate was similar to the background signal, indicating that no bacteria survived on the surface of the plate. SEM analysis revealed *S. aureus* spherical bodies and bacterial biofilm on the surface of the non-coated PEEK plate (Fig. 3C), but not on the PEEK-Ag^+^ plate (Fig. 3D).

### Antibacterial effect in vivo

**Figure 4:**
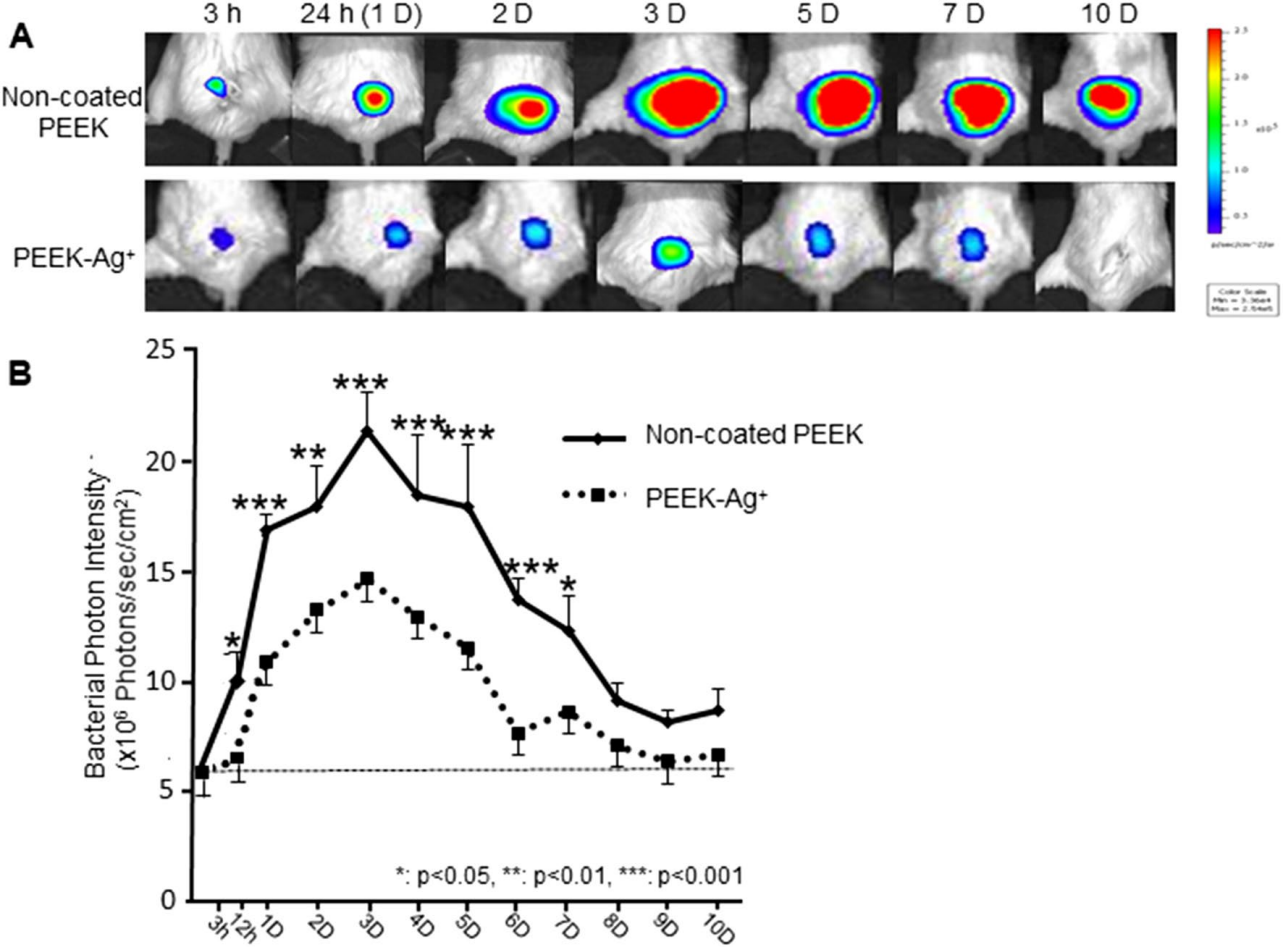
Sequential analysis of the bacterial bioluminescence in the mouse soft tissue infection model. A non-coated PEEK or PEEK-Ag^+^ plate was placed into the superficial gluteus muscle (SGM) of mouse, followed by inoculation of a bioluminescent strain of *S. aureus.* The bacterial photon intensity (PI) was sequentially measured by BLI at 3, 12, and 24 h, and then each day until 10 days after the operation (**A**). The non-coated PEEK revealed that the mean bacterial PI increased at 12 h, peaked on Day 3, and then gradually decreased until Day 8 after the inoculation. However, the signals were still high on Day 10. In contrast, in the PEEK-Ag^+^ group, the mean bacterial signals increased on Day 1 and peaked on Day 3, but then dramatically decreased after Day 8. The bacterial signal dramatically disappeared on Day 10, to the background level. The mean PI at each time point between 12 h and 7 days in the PEEK-Ag^+^ group was significantly lower than that observed in the non-coated PEEK group (12 h: *p* = 0.0261, Day 1: 0.0004, Day 2: 0.0035, Day 3: 0.0005, Day 4: 0.0003, Day 5: 0.0002, Day 6: 0.0008, Day 10: 0.0186) (**B**). Results shown are means ± standard errors of the mean (SEM).

We previously showed that the bacterial PI is directly proportional to the number of bacterial CFUs in vivo (*R*^2^ = 0.988)^19^. We also demonstrated a significant correlation between the number of pixels of gram-positive area on the superficial gluteus muscle (SGM) tissue sections and the bacterial PI in vivo (*R*^2^ = 0.979)^19^. In the present study, a strong bacterial bioluminescent signal was detected only at the surgical site of the SGM, and the surrounding tissue was free of infection for 10 days after surgery (Fig. 4A). Sequential analyses of the bacterial luminescence in the non-coated PEEK group revealed that the mean bacterial PI in the SGM increased 12 h after inoculating *S. aureus* into the left SGM, peaked on Day 3 (21.34 ± 2.77 × 10^6^ PI), and then gradually decreased until Day 8. However, the PI was still high on Day 10 (Day 5: 17.97 ± 1.59 × 10^6^ PI, Day 8: 9.10 ± 0.95 × 10^6^ PI, Day 10: 8.70 ± 0.66 × 10^6^ PI) (Fig. 4B). In contrast, in the PEEK-Ag^+^ group, the mean bacterial PI increased on Day 1 and peaked on Day 3 (14.64 ± 2.36 × 10^6^ PI), and then dramatically decreased after Day 8 (7.08 ± 1.02 × 10^6^ PI) (Fig. 4B). The bacterial signal dramatically disappeared (Fig. 4A), and the mean PI after Day 10 was 6.69 ± 0.67 PI, which is the same as the background level (Fig. 4B). It is suggested that no bacteria survive and the infection has subsided in the PEEK-Ag^+^ group after Day 10. At each time point from 12 h to 7 days, the mean PI in the PEEK-Ag^+^ group was significantly lower than that observed in the non-coated PEEK group (12 h: *p* = 0.0261, Day 1: *p* = 0.0004, Day 2: *p* = 0.0035, Day 3: *p* = 0.0005, Day 4: *p* = 0.0003, Day 5: *p* = 0.0002, Day 6: *p* = 0.0008, Day 10: *p* = 0.0186) (Fig. 4B).

**Figure 5:**
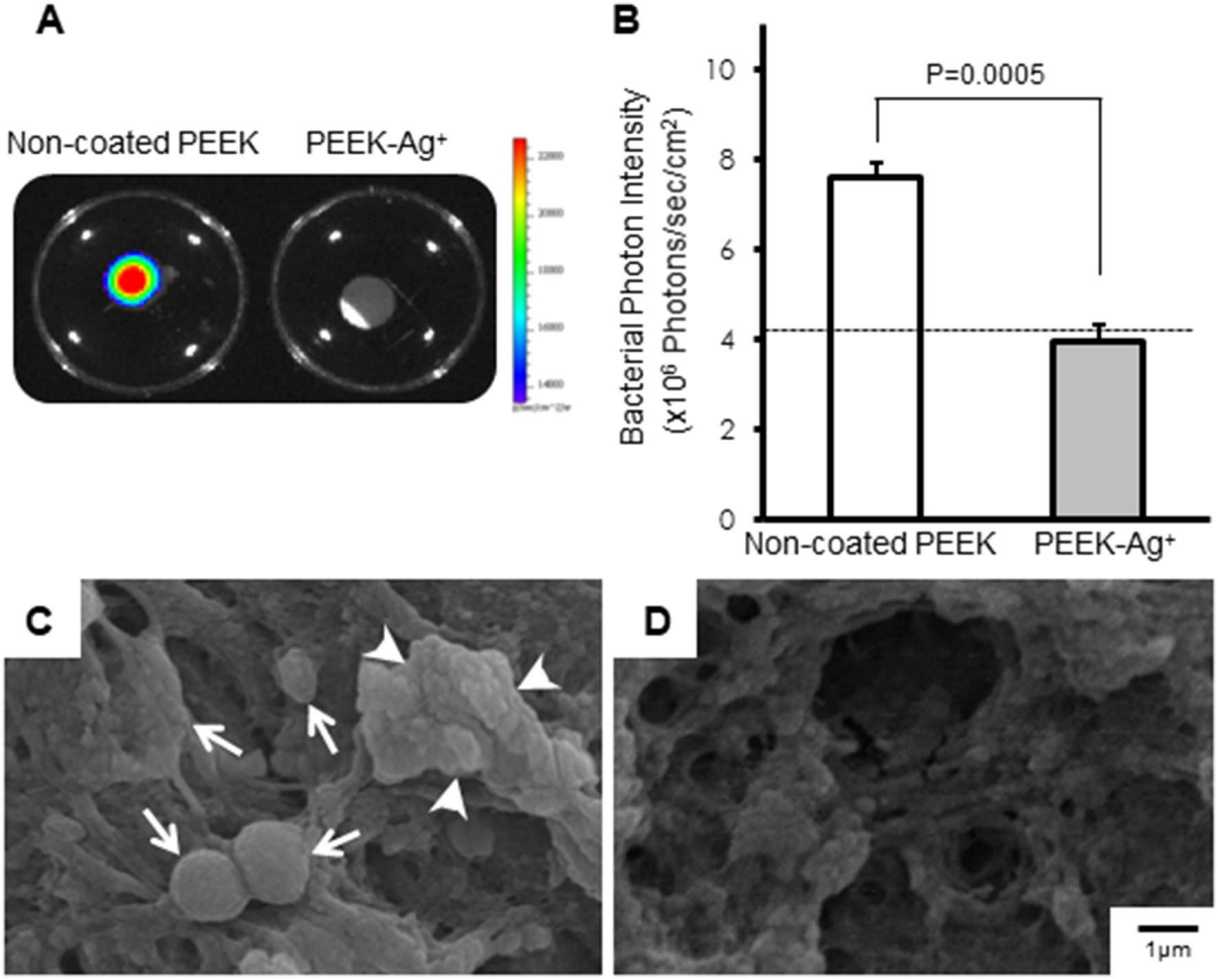
Optical and SEM images of PEEK plates removed from the infection mouse model. On Day 14, implanted PEEK plates were removed from the SGM and washed 3 times in phosphate buffered saline (PBS). The bacterial bioluminescent signals were then captured by BLI. A strong signal was observed on the surface of the non-coated PEEK, but not on the PEEK-Ag^+^ (**A**). The signal on the non-coated PEEK was significantly greater than that on the PEEK-Ag^+^ (*p* = 0.0005) (**B**). The microstructures of the surface of these PEEKs were analyzed by SEM. *S. aureus* (arrows; **C**) and its biofilm (arrowheads; **C**) were seen on the surface of the non-coated PEEK, but not on the PEEK-Ag^+^ (**D**). Results shown are means ± standard errors of the mean (SEM).

On Day14, the implanted PEEK plates were removed from the SGM, washed 3 times in PBS, and the bacterial bioluminescent signals were captured by BLI. A strong signal was observed on the surface of the non-coated PEEK, but not on the PEEK-Ag^+^ (Fig. 5A). The mean PI of the PEEK-Ag^+^ was significantly lower than that of the non-coated PEEK (3.92 ± 0.40 PI vs. 7.59 ± 0.35 PI: *p* = 0.0005) (Fig. 5B). The microstructures of the surface of these PEEKs were also analyzed by SEM. *S. aureus* and its biofilm were seen on the surface of the non-coated PEEK (Fig. 5C), but not on the PEEK-Ag^+^ (Fig. 5D).

**Figure 6:**
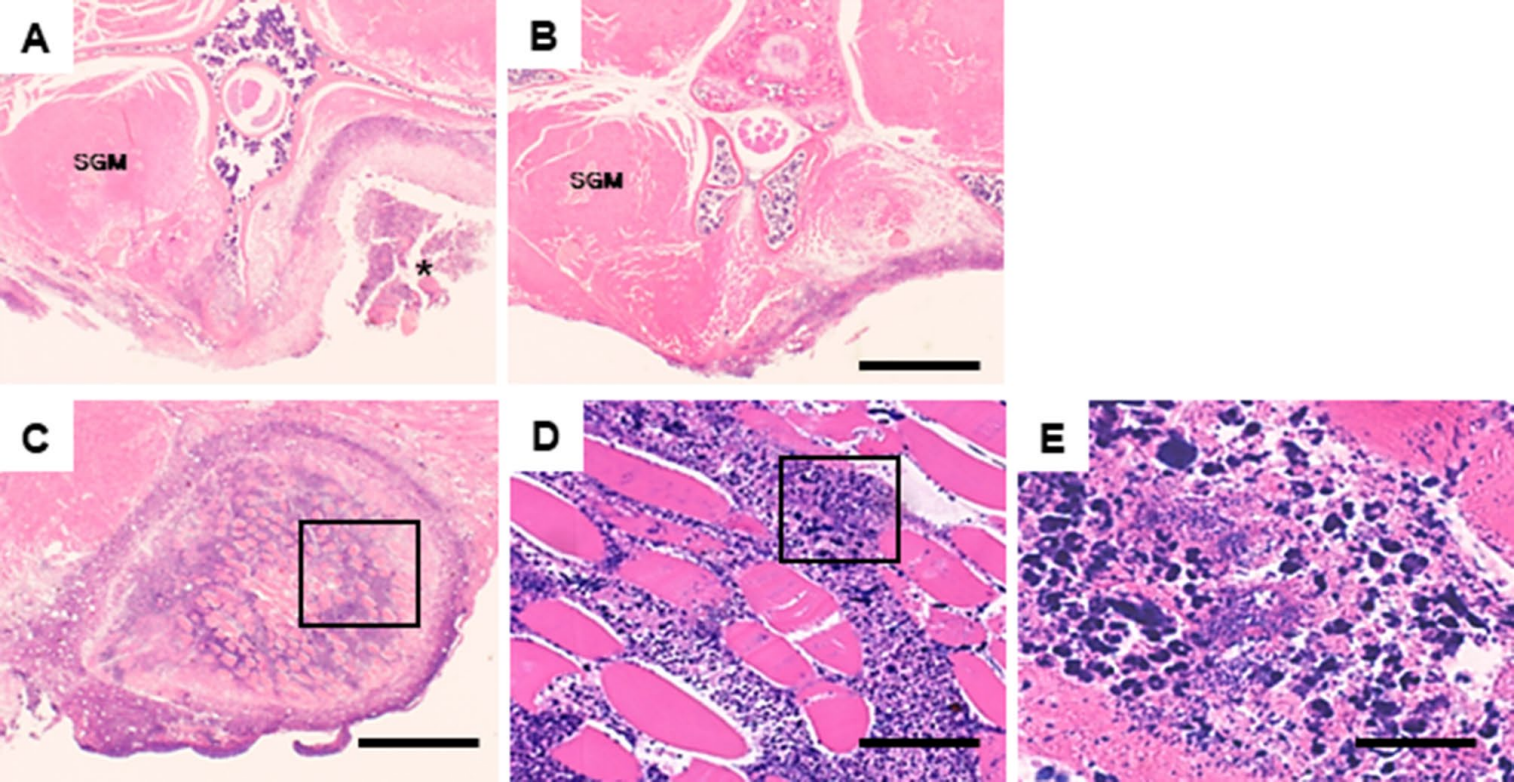
Histopathology of the SGMs and spine from the mouse implanted with PEEK. Mice implanted with non-coated PEEK or PEEK-Ag^+^ plates were sacrificed at day 14 and the bilateral SGMs and spine from the mice were histologically examined (**A**–**E**). A large abscess (*) was seen at the surgical site in the non-coated PEEK-treated mouse (**A**), but not in the PEEK-Ag^+^-treated one (**B**). The contralateral SGMs were intact in both groups. The images in (**D**) and (**E**) images are higher-power views of the boxed areas in (**C**) and (**D**), respectively. Bacterial clots were found, as well as infiltrating inflammatory cells such as neutrophils and proliferating fibroblasts around the muscle in the non-coated PEEK group (**C**–**E**). Bars = 2000 µm (**A** and **B**), 1000 µm (**C**), 400 µm (**D**), 200 µm (**E**).

Histologically, a large abscess was seen at the surgical site of the non-coated PEEK-containing mice (Fig. 6A), but not of the PEEK-Ag^+^-containing mice (Fig. 6B). The contralateral SGMs were intact in both groups. Bacterial colonies were found, as well as inflammatory cells such as neutrophils and proliferating fibroblasts grown around the necrotic muscle fibers in the non-coated PEEK-containing group (Fig. 6C–E). In contrast, the histological features of infection were strongly reduced in the treated muscle in the PEEK-Ag^+^-containing group (Fig. 6B).

### Validation of biomechanical stability

**Figure 7:**
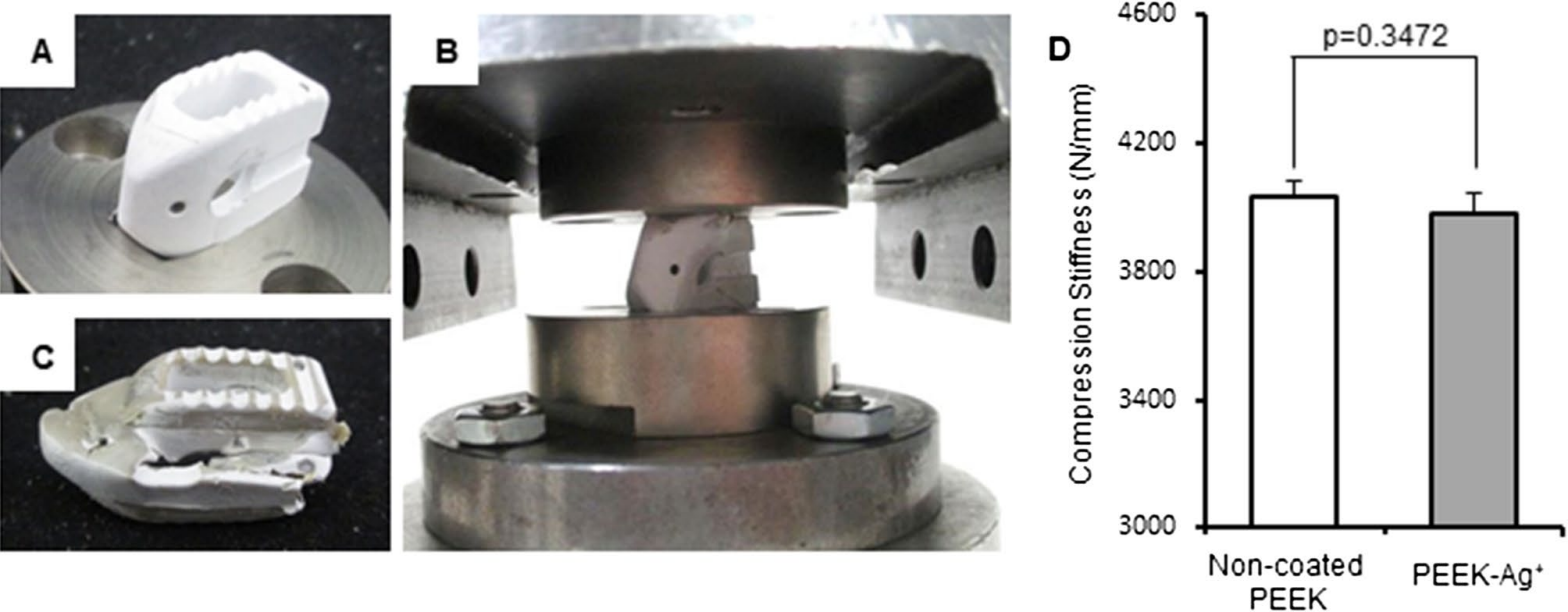
Validation of biomechanical stability. Intervertebral spinal cages were prepared by Ortho Development (VUSION). A biomechanical compression test was performed for non-coated PEEK and PEEK-Ag^+^ cages (**A**–**C**). The average compression stiffness of the non-coated PEEK and PEEK-Ag^+^ cages was 4034 ± 49 N/mm and 3980 ± 65 N/mm, respectively (**D**). There was no significant difference between the two groups (*p* = 0.347). Results shown are means ± standard errors of the mean (SEM).

Compression tests were performed for the non-coated PEEK and PEEK-Ag^+^ (Fig. 7). Failure represented by plastic deformation of the cages was observed (Fig. 7B,C). The average compression stiffness of the non-coated PEEK and PEEK-Ag^+^ cages was 4034 ± 49 N/mm and 3980 ± 65 N/mm, respectively (Fig. 7D). There was no significant difference between the results for the two groups (*p* = 0.3472).

## Discussion

Prosthetic joints and spinal implants are widely used as orthopedic surgical procedures. In recent years, ceramics and polyethylene have become broadly accepted as radiolucent alternatives to metallic biomaterials in the orthopedic community. Synthetic resin materials have also been used for these devices, with PEEK being the most popular polymer^28^. PEEK was developed in the 1980s^29^, and has gained notice as a thermoplastic polymer material, especially in orthopedics and trauma applications, since the 1990s^30–32^.

The number of IAI cases has been increasing. IAI is typically caused by microorganisms growing in structures known as biofilms^33^. In orthopedic cases, IAI induces progressive inflammation and destructive bone changes. To prevent IAI, various surface modifications of implants have been tested, including antibiotics^34^, quaternary ammonium compounds^35^, and silver coatings^36^, with mixed clinical success. A silver-coated antibacterial intravascular catheter^12^ and silver-alloy-coated urethral catheter^11^ have been reported. Vascular grafts using chitosan^37^ or denture stomatitis treatment using an organic composite antibacterial agent^10^ are already being applied clinically. However, no antibacterial polymer implant had been demonstrated to date. Although various silver-coated implants have been shown to have antibacterial effects in vitro^38,39^, their in vivo results are not convincing^40,41^. Silver is thought to exert antibacterial effects by mediating the generation of reactive oxygen species in the presence of oxygen^42^. Therefore, to maintain antibacterial activity in vivo, the silver should remain in an ionic state^43^. We have previously developed an chelated ionic silver coating through a complex of HAp and IP6 on a metal material, which provided a strong and stable antibacterial effect both in vivo and in vitro without cell toxicity^14^. We have recently applied our coating method to PEEK and, successfully produced a preliminary ionic silver chelation coating on a PEEK substrate^15^. Silver coatings are typically produced using high-temperature processes, such as the plasma spray method^44^, that heat the device to several thousand degrees Celsius. Although these methods cannot be used for plastics and ceramics, our ionic-silver chelating technique is carried out at much lower temperatures, and enables to bind the silver ions strongly by IP6 chelation. In the present study, we demonstrated an optimal processing of surface modification of PEEK for our bacteria-resistant coating and observed a potent antibacterial effect both in vitro and in vivo. To the best of our knowledge, this is the first report describing a coating method for a polymer that provides strong antibacterial activity both in vitro and in vivo.

PEEK has an inherent chemical inertness as well as stability at high temperatures, resistance to radiation damage, and strength. However, it is difficult to modify chemically with drug solutions, and is dissolved only by highly concentrated H_2_SO_4_^45,46^. When treated with highly concentrated H_2_SO_4_, the structure of PEEK surface changes^47^ to form a porous layer. The pore size is reported to be suitable for the migration of osteoblasts^48^. According to these previous observations, we first modified the PEEK surface to create pores that could act as a binding surface for the coating process. We also determined the optimal treatment time for the highly concentrated H_2_SO_4_. SEM observation confirmed that the immersion into SBF created a coating film of HAp aggregates on the surface of the porous layer. The pore diameter and existence of HAp film indicated that the biocompatibility of this fabricated PEEK would be high, and thus useful as an implant material. We then successfully applied immobilized Ag^+^ ions to the HAp film by the tight chelate-bonding action of IP6 on the surface of the fabricated PEEK. The results of a peeling test indicated that the pores on the PEEK surface produced by the H_2_SO_4_ treatment acted as a scaffold to hold the HAp tightly to the PEEK. Moreover, EDX showed that the Ag^+^-immobilized HAp consisted of Ca, P, O, and C, as well as Ag. In addition, ICP-AES indicated that the Ag^+^ ion content in the HAp film was dependent on the AgNO_3_ concentration used, indicating that the antibacterial ability of the Ag^+^-immobilized HAp can be easily controlled by changing the concentration of AgNO_3_ solution during the preparation. In the biomechanical analysis using a spinal interbody cage, PEEK-Ag^+^ show a similar stability as non-coated PEEK. Thus, our coating method is easy and simple at a lower cost and provides tight attachment of HAp on the PEEK without any biomechanical degradation.

In our in vitro antibacterial evaluation, PEEK-Ag^+^ showed a strong antibacterial effect after a 24-h incubation with bacteria*.* Surprisingly, no live bacterial signals were detected in the PEEK-Ag^+^ sample, indicating that the Ag^+^ immobilized on HAp provided sterilizing activity as well as an antibacterial effect. SEM analysis showed *S. aureus* spherical bodies and bacterial biofilm on the surface of non-coated PEEK, while no bacteria or bacterial biofilm were seen on the PEEK-Ag^+^. Demonstration of an antibacterial effect in vivo is very important from the standpoint of clinical application. We previously developed a mouse model of soft-tissue infection using BLI, which enables the noninvasive, real-time monitoring of bacterial growth^19^. The SGM is a large muscle with thick muscular fascia, and is located near the back surface of a mouse’s body. The bacterial signal using this model is typically observable for a long time (up to 6 weeks) if there is no leakage from the fascia. In the present study, we could observe the bacteria and antibacterial effects for at least 14 days using this model. Sequential analyses of the bacterial luminescence in non-coated PEEK-containing animals showed that the mean bacterial PI in the SGM increased 12 h after *S. aureus* inoculation into the left SGM, peaked on Day 3, and then gradually decreased until Day 8. However, the PI remained high even beyond Day 21 (data not shown). In contrast, in the PEEK-Ag^+^-containing animals, the average bacterial PI increased on Day 1, peaked on Day 3, and then dramatically decreased after Day 8. The bacterial signal disappeared by Day 10, to the background level. On Day 21, no recurrence of infection was observed in any of these animals (data not shown). On the PEEKs removed from the animals, a strong live bacterial signal was observed on the non-coated PEEK, but not on the PEEK-Ag^+^. In addition, *S. aureus* and its biofilm were seen on the surface of the non-coated PEEK, but not on PEEK-Ag^+^. In the PEEK-Ag^+^ group, a xenobiotic reaction and cell toxicity toward the host tissue were recognized, although the formation of ulceration was clearly suppressed. Bacterial clots could also not be confirmed, and part of the SGM was preserved. These observations indicated that the Ag^+^ ions not only decreased the bacterial growth but also killed bacteria, thereby inhibiting biofilm formation. PEEK’s lack of toxicity was demonstrated in 1990^49^. The toxicity of Ag^+^ ions is well-known, and is reported to induce argyria, leukemia, and liver/kidney dysfunction in humans at serum levels greater than 300 ppm or systemic levels greater than 4–6 g. We previously showed that the toxicity of the coating treated with 5 mmol·dm^−3^ AgNO_3_ was less than 25%, which is acceptable according to Food and Drug Administration (FDA) standards^14^. According to the results in the current study, 5 mmol·dm^−3^ AgNO_3_ may be a suitable concentration in terms of toxicity.

There are several hypotheses about the antibacterial action of Ag^+^ ions. For example, oxygen changes to active oxygen by a catalytic action of Ag^+^, and the active oxygen may destroy the cell membrane; or, Ag^+^ ions are so small that they easily pass through the cell membrane and inhibit enzymes inside the cell. A generally accepted theory is that Ag^+^ ions inactivate membrane proteins, and bind with the bacterial DNA, disrupting its replication by replacing the H^+^ ions acting as cross-linkers in the DNA strands, thereby impairing the ability of ribosomes to transcribe messenger RNA into vital proteins required for bacterial cell function^42,50^.

In conclusion, we have developed a polymer coated with Ag^+^ ions in a stable and uniformly dense manner. This is the first demonstration of Ag^+^-coated polymer implant that prevents the formation of bacterial biofilms and provides significant antibacterial effects in vitro and in vivo. This new Ag^+^ ion coating method can be applied to various medical devices to prevent implant-associated infection. For clinical use, PEEK and the solutions used to prepare PEEK-Ag^+^ are inexpensive, and could easily be processed in a heating device or incubator. Our finding that PEEK-Ag^+^ elicited an antibacterial effect in a model with a completely buried polymer implant for an extended period of time represents an important milestone in antibacterial implants, and may be a promising method for preventing IAI in human patients.

## Methods

All methods were performed in accordance with the relevant guidelines and regulations. The study was carried out in compliance with the ARRIVE guidelines, and all animal experiments were approved by the Animal Care and Use Committee of Keio University.

### Preparation of PEEK coated with immobilized Ag^+^ ions (PEEK-Ag^+^)

PEEK plates (φ5.0 mm × 1 mm-thick; 450 G; VICTREX, UK) were prepared for each experiment. The surface of the plate was roughened using a sandblasting machine (BUEHLER, IL, USA) with silicon carbide paper (#600; SANKYO RIKAGAKU, Japan), followed by ultrasonic washes with pure water and ethanol. The PEEK surface was then immersed into 98% concentrated H_2_SO_4_ (WAKO CHEMICAL Japan) for various periods of time (1, 3, 5, 10, and 15 min) to produce a binding surface. After dry heat treatment at 110 °C for 30 min, a soft solution process was applied consisting of simulated body fluid (SBF), urea, and urease. Specifically, urea in SBF was adjusted to 2.0 mol/dm^3^, and applied to the PEEK surface, followed by a 1.0 mass % urease solution, at 0.0534 cm^3^ per 5 cm^3^ of PEEK surface. The material was heated at 36.5 °C for 24 h, followed by its reimmersion in SBF. The material was then placed at 50 °C, and the SBF was exchanged every 48 h, for a total of 3 times. By this treatment, HAp film was produced on the surface of the PEEK substrate. The substrate was then immersed in IP6 solution (1000 ppm) at 50 °C for 24 h, followed by 10 min in AgNO_3_ solution (5 mmol·dm^−3^) at 25 °C, according to our previous protocol^14^.

### Microstructure and component analyses of PEEK-Ag^+^

To determine the optimal immersion time of the H_2_SO_4_ treatment, PEEK samples treated by H_2_SO_4_ for various durations were cut by a diamond cutter, and the cross-sectional surface was subjected to microstructure analysis using SEM (JSM-6390LA, JAPAN ELECTRON OPTICS LABORATORY, Tokyo, Japan). The average thickness of the modified layer of the PEEK surface was measured (N = 3). The surface of the PEEK-Ag^+^ was also observed by SEM. For component analysis, the PEEK-Ag^+^ was subjected to EDX and ICP-AES. In addition, the qualitative bonding strength between PEEK and HAp was analyzed as previously reported by Tretinnikov et al^51^. Briefly, a piece of Scotch tape (#810: 3 M Japan, Tokyo, Japan) was placed on PEEK-Ag^+^ or on PEEK subjected to the Ag^+^ coating process without H_2_SO_4_ treatment, and then peeled away to examine the strength of adhesion of the HAp film coating.

### Antibacterial effect of PEEK-Ag^+^

#### Bioluminescent bacteria

A bioluminescent strain of *S. aureus* Xen-29 was obtained from CALIPER LS Co. (Hopkinton, MA, USA) and cultured in Luria Bertani medium (SIGMA-ALDRICH Co., St. Louis, MO, USA) at 37 °C, under ambient aeration with gentle agitation. The bacteria were selectively grown on medium containing 200 µg/ml kanamycin. *S. aureus* Xen-29, derived from the parental strain ATCC 12600, has a stable copy of a modified *Photorhabdus luminescens luxABCDE* operon, which encodes enzymes responsible for the luminescent reaction. Bacterial bioluminescence requires no added substrate to generate light; the organism constitutively emits a bioluminescent signal as long as it is alive. The luminescent reaction can generate visible light in the presence of oxygen and, typically, ATP as a source of energy. The bacteria samples were frozen and stored at − 80 °C. The samples were thawed at 4 °C for one hour prior to each experiment. Typically, the bacterial viability was maintained at 4 °C for approximately 5 h after thawing^18^.

#### Antibacterial effect of PEEK-Ag^+^ in vitro

Non-coated PEEK and PEEK-Ag^+^ plates (N = 3 each) were immersed into 500 µl of medium containing 5 × 10^7^ CFU bioluminescent *S. aureus*, and incubated at 37 °C for 24 h. After 3 10-min washes in PBS, the bacterial signal on the PEEK surface was captured by the BLI system and the surface of each PEEK sample was also observed by SEM as described below.

#### Antibacterial effect of PEEK-Ag^+^ in vivo

To evaluate the antibacterial effect of PEEK-Ag^+^ in vivo, we used a reproducible and stable mouse soft-tissue infection model that we reported previously^19^. BALB/c adult male mice (12 weeks old, 20–25 g) purchased from SANKYO LABO SERVICE CORPORATION INC. (Shizuoka, Japan) were used. The mice were anesthetized with an intraperitoneal administration of 50 mg/kg pentobarbital^18,19^. A skin incision was made at the left side of the spine, and the SGM was exposed. A non-coated PEEK or PEEK-Ag^+^ plate was placed into the SGM (N = 5 each); then, a bioluminescent strain of *S. aureus* (1 × 10^11^ CFU/ml) in 5 µl of medium was inoculated into the muscle. The muscle fascia was sutured tightly to prevent bacterial leakage. The skin wound was also tightly sutured. As a preliminary experiment of biological reaction of implanted PEEK, no adverse reactions and complications caused by xenobiotics, such as redness, pus formation, fever were observed (data not shown). The advantages of this model are that (1) the SGM is the largest muscle in mice, and (2) the thick fascia confines the growing bacteria within a large muscle volume, making it suitable for observation over long periods of time^19^. We previously showed that the bacterial bioluminescence, observable by BLI at the inoculated region was stable for at least 4 weeks. The bacteria and abscesses were confined to the left SGM even 4 weeks after inoculation, and non-SGM tissue specimens from the inoculated animals did not show any leaked bacteria^19^.

In the present study, the bacterial PI was sequentially measured by BLI at 3, 12, and 24 h, and then each day until 10 days after the operation. During the BLI measurement, the mice were anesthetized via inhalation of isoflurane mixed with oxygen. Fourteen days after the inoculation of bacteria and PEEK plates, the PEEK plate was collected from the muscle. After three 10-min washes in PBS, the bacterial signal on the PEEK surface was captured by the BLI system as described below. The surface of each PEEK was also observed by SEM.

### Bioluminescence imaging (BLI)

A CALIPER LS-IVIS Lumina (SUMMIT PHARMACEUTICALS INTERNATIONAL Co., Tokyo, Japan) cooled CCD optical macroscopic imaging system was used for the BLI^52^. Photon emissions of the bacterial bioluminescent signal were captured, converted to false color photon count images, and quantified with Living Image version 3.0 software (CALIPER LS Co., Hopkinton, MA, USA). The bacterial PI was expressed as photon flux, in units of photons/sec/cm^2^/steradian. We previously showed that the bacterial PI in this model is strongly correlated with the number of bacteria both in vitro and in vivo^17–19^.

To quantify the bacterial luminescence in vitro, non-coated PEEK and PEEK-Ag^+^ plates were separately immersed with bacteria for 24 h in a microtube (Eppendorf tube 1.5 ml, EPPENDORF Japan, Tokyo, Japan). The plates were then removed and washed. For imaging analyses, regions of interest (ROIs) were defined in the extract medium in the microtubes and on the plates. In the in vivo animal experiment, the bacterial PI in an ROI at the left SGM was measured at 3, 12, and 24 h, and then every day until day 10. The surfaces of the non-coated PEEK and PEEK-Ag^+^ plates removed from the SGM were also analyzed. The luminescence signal was quantified within the defined ROIs on the plates and in the inoculated areas.

### Histopathological analysis

Fourteen days after surgery, mice were subjected to the last observation by BLI and sacrificed under intraperitoneal administration of a high dose of pentobarbital. The bilateral SGMs and spine of the mice that had contained a coated PEEK or PEEK-Ag^+^ plate were isolated en block and analyzed by histopathology. The samples were fixed in 4% paraformaldehyde and demineralized with ethylenediaminetetraacetic acid. The samples were then embedded in paraffin, cut into 5-µm-thick sections, and stained with hematoxylin and eosin.

### Scanning electron microscopy (SEM)

The cross-sectional surface of the H_2_SO_4_-treated PEEKs, the surface of PEEKs from various treatment steps, and the PEEKs removed from animals were analyzed by SEM (JSM-6390LA, JAPAN ELECTRON OPTICS LABORATORY). Each sample was fixed for 24 h in 2% PFA and 2% glutaraldehyde at 4 °C. After drying, the sample was mounted on metal stubs, metal-coated with platinum-palladium using an ion-coater, and observed by SEM at an accelerating voltage of 5 kV.

### Validation of biomechanical stability

PEEK cages for lumbar fusion procedures were received from Ortho Development (VUSION, Salt Lake City, UT, USA). Non-coated PEEK and PEEK-Ag^+^ samples were prepared (N = 4). All materials were clean cages without biofilm. Two stainless steel test blocks were machined to hold the implant, based on drawings received from ORTHO DEVELOPMENT (see Fig. 7A,B). The geometry of the blocks matched that of the implants to keep them stable during the test. The test blocks had 3-mm-deep pockets, and the resulting intradiscal height was 11 mm. Static tests were performed on a Satec Model UTC, S/N 1412. Force was measured using a 1220ACK-50 K load cell and S/N 293153A. The linear encoder LI-24, S/N 0219-0000 was used to measure displacement. Eight total test specimens were assembled between the two prepared metal test blocks by Accutek engineers. The test assemblies were mounted and aligned in the test frames using the Compression Testing Configuration. Axial load was applied in the Z direction at 25 mm/min until failure to maintain load or limitation of the test frame was reached. Failure was defined if the implant showed plastic deformation.

### Statistical analysis

The different time periods of H_2_SO_4_ treatment for modifying the PEEK surface, and time-course changes in the bacterial PI in animals were analyzed by one-way ANOVA followed by Fisher’s PLSD post-hoc test. The antibacterial effect in the in vitro analysis of PEEK plates, and the biomechanical analysis of PEEK cages were analyzed by Student’s t-test. Statistical analyses were performed with SPSS II software (IBM-SPSS, Tokyo, Japan), and a probability value less than 0.05 was considered significant.
